# Studying the connection between SF3B1 and four types of cancer by analyzing networks constructed based on published research

**DOI:** 10.1038/s41598-023-29777-5

**Published:** 2023-02-15

**Authors:** Asmaa Samy, Mehmet Kemal Ozdemir, Reda Alhajj

**Affiliations:** 1 The Graduate School of Engineering and Natural Science, Istanbul Medipol University, Istanbul, Turkey; 2 School of Engineering and Natural Science, Istanbul Medipol University, Istanbul, Turkey; 3 Department of Computer Science, University of Calgary, Calgary, AB Canada; 4 Department of Heath Informatics, University of Southern Denmark, Odense, Denmark

**Keywords:** Breast cancer, Biomarkers

## Abstract

Splicing factor 3B subunit 1 (SF3B1) is the largest component of SF3b protein complex which is involved in the pre-mRNA splicing mechanism. Somatic mutations of SF3B1 were shown to be associated with aberrant splicing, producing abnormal transcripts that drive cancer development and/or prognosis. In this study, we focus on the relationship between SF3B1 and four types of cancer, namely myelodysplastic syndrome (MDS), acute myeloid leukemia (AML), and chronic lymphocytic leukemia (CLL) and breast cancer (BC). For this purpose, we identified from the Pubmed library only articles which mentioned SF3B1 in connection with the investigated types of cancer for the period 2007 to 2018 to reveal how the connection has developed over time. We left out all published articles which mentioned SF3B1 in other contexts. We retrieved the target articles and investigated the association between SF3B1 and the mentioned four types of cancer. For this we utilized some of the publicly available databases to retrieve gene/variant/disease information related to SF3B1. We used the outcome to derive and analyze a variety of complex networks that reflect the correlation between the considered diseases and variants associated with SF3B1. The results achieved based on the analyzed articles and reported in this article illustrated that SF3B1 is associated with hematologic malignancies, such as MDS, AML, and CLL more than BC. We found that different gene networks may be required for investigating the impact of mutant splicing factors on cancer development based on the target cancer type. Additionally, based on the literature analyzed in this study, we highlighted and summarized what other researchers have reported as the set of genes and cellular pathways that are affected by aberrant splicing in cancerous cells.

## Introduction

Cancer is a multi-factorial disease, caused by a combination of genetic, epigenetic, environmental, and behavioral factors^1,2^. Somatic mutations of splicing factors are considered among the drivers of cancer development. They cause aberrant splicing that produces abnormal transcripts, which are either degraded via nonsense-mediated mRNA decay (NMD) mechanism that reduces proteome content of the cell^3^, or they are translated into aberrant proteins^4–6^. Previous studies demonstrated that these abnormal proteins are involved in many tumor biological processes such as proliferation, metastasis, and apoptosis^7^.

The Global Cancer Observatory (GLOBOCAN) 2018 database reported that cancer, in general, is the second cause of death, leading to 9:6 million deaths in 2018. Lung, breast, and colorectal cancer have the highest incidence rates among all types of cancer. Additionally, they are also placed among the top lethal cancers. Across 185 countries, lung, breast, and colorectal cancer types have been attributed to one-third of the cancer incidents and mortality for both genders^8^. Precursor messenger RNA (pre-mRNA) splicing is a critical mechanism of RNA metabolism, where introns are excised from pre-mRNA and only coding exons are translated into an amino acid sequence. It is executed by the spliceosome, which is a dynamic complex ribonucleoprotein (RNP). RNP is comprised of five small nuclear RNPs (snRNPs) (U1, U2, U4, U5, and U6) and many other proteins^9^. In the step of 3′ splice site (SS) cleavage and exon ligation, SF3b complex is essential for accurate recognition of branch point (BP) and selection of splicing site. It is a multi-protein component of U2 snRNP. It consists of seven subunits: SF3B1, SF3B2, SF3B3, SF3B4, SF3B5, SF3B6, and SF3B7^10^.

Splicing factor 3B subunit 1 (also known as SF3B1 or SF3b155) is the largest component of SF3b protein complex^11,12^. Realized as a critical component of the splicing machinery, SF3B1 catalyzes the removal of introns from precursor messenger RNA (mRNA). It has 20 repeats of HEAT domain (HD)^13^, which contains most of the mutations associated with various cancer types. Some of the most reported diseases are myelodysplastic syndrome (MDS)^13^, chronic lymphocytic leukemia (CLL)^14^, uveal melanoma^15^, and breast cancer as a solid tumor^16^.

Most splicing related studies are based on experimental approaches. For instance, in 2015, Papasaikas et al. used an experimental approach to analyze the impact of knocking down spliceosome components on cell proliferation and apoptosis. Then, they used this information to reconstruct a network of functional interactions among splicing factors to highlight therapeutic targets. In 2019, Borišek et al. studied subcomplex of the spliceosome B^act^ state from Saccharomyces cerevisiae using molecular dynamics (MD) simulations^17^. Away from these two articles and other few studies, the rest of the literature mostly studied SF3B1 alone or in combination with few splicing factors. However, proteins function within multicomponent proteins, and all these large proteins act in complex protein association networks for different cellular pathways.We have analyzed the literature from pubmed for the period 2007–2018. We tried to concentrate only on articles written by researchers who studied SF3B1 in connection with MDS, AML, CCL and BC. Our target is to find out how SF3B1 has influenced the research community as a driver of some types of cancer in addition to its other roles reported in the literature. However, the latter roles are outside the scope of the study described in this article, though essential they may be covered in a separate future article..

To achieve the goal of this study, several publicly available databases, including COSMIC (https://cancer.sanger.ac.uk/cosmic), DisGeNET (https://www.disgenet.org), and HGNC (https://www.genenames.org) were used to extract some relevant information mostly related to SF3B1 and its variants in association with the investigated four types of cancer. In particular, the association between each of the four diseases and SF3B1 or its family have been separately identified based on COSMIC database. COSMIC is the world’s largest and most comprehensive resource for exploring the impact of somatic mutations in human cancer. The gene-disease association score is computed by considering DisGeNET database. The process of computing the latter score considers the number and type of the sources (level of curation, organisms), and the number of publications supporting the association, leading to a value in the range between 0 to 1. The whole process of computing the score is described in https://www.disgenet.org/dbinfo#score.

DisGeNET is a discovery platform which contains one of the largest publicly available collections of genes and variants associated to human diseases. Families to which SF3B1 belongs were identified using HGNC database, which is considered as the resource for approved human gene nomenclature. Concentrating on SF3B1 in connection to the mentioned four types of cancer, we built various gene networks to provide a better understanding of SF3B1 association with cancer. Explicitly speaking, DisGeNET was used to validate the data from COSMIC database, and accordingly, networks reflecting gene-disease associations and variant-disease associations were constructed and analyzed, among others. From these networks, various aspects related to SF3B1 were highlighted. Mutations of SF3B1 were located and its links to the investigated types of cancer were studied to realize the increased interest in this regard.

## Results and discussion

In this section, COSMIC SF3B1 data were analyzed from a historical perspective (spatial and temporal) for two purposes: (i) showing the trend of interest for studying SF3B1 as a gene associated with MDS, AML, CLL, and breast, and (ii) listing all countries which studied this association. Additionally, the count of distinct “PubMed_PMID" for each gene associated with every one of the considered diseases was retrieved in descending order and then was compared with SF3B1. Various networks have been constructed and analyzed based on information extracted from some publicly available databases which have been demonstrated effective in this context. The results combined with the related discussion are reported in this section.

### SF3B1 is linked to hematologic cancer more than breast cancer in the literature

**Figure 1:**
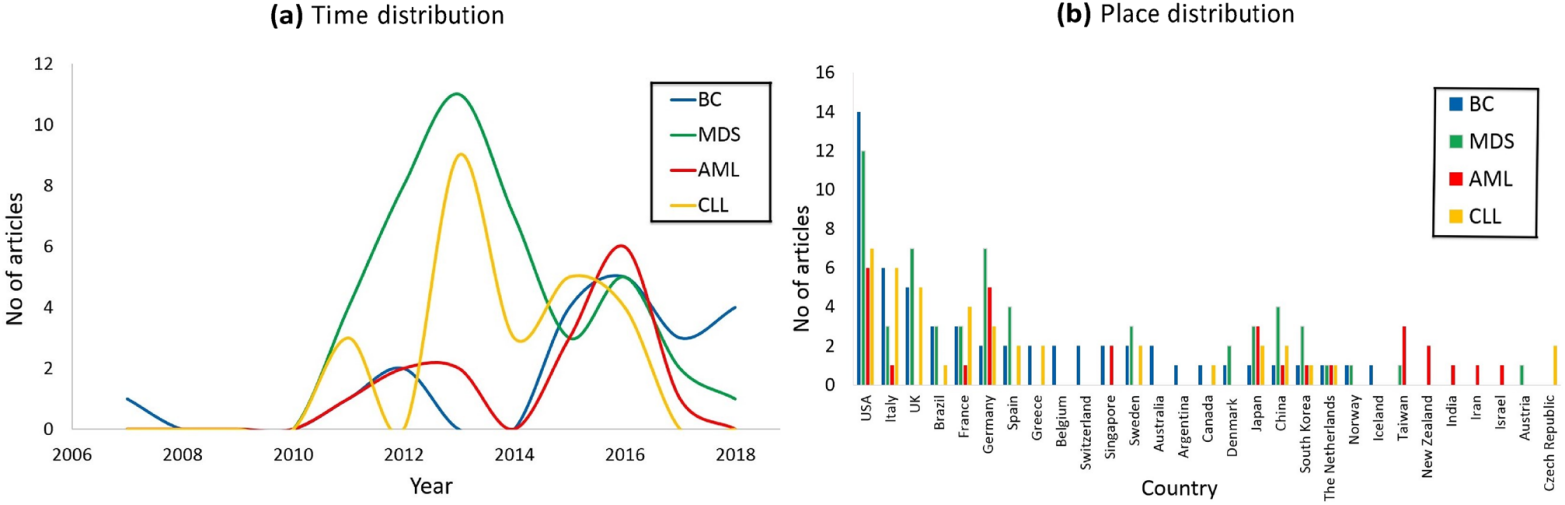
Distribution plots of (**a**) time line of articles that included SF3B1 and one or more associated type of cancer from 2007 to 2018. (**b**) All countries that performed these studies in the given time period. Breast cancer (BC), MDS, AML, and CLL are colored in blue, green, red, and orange, respectively.

COSMIC data of blood and breast cancers illustrate that SF3B1 was reported 100 times in the literature starting from 2007 until 2018 as shown in Fig. 1a which reports the numbers of Pubmed research articles that mentioned SF3B1 in connection with the considered four types of cancer during the period 2007 to 2018. For each category of the latter articles which link SF3B1 to the four types of cancer, Fig. 1b reports their distribution by country. In 2007, SF3B1 and breast cancer were mentioned together in one article. From 2008 to 2010, there is no publication where SF3B1 is associated with any of the four types of cancer. In 2011, the number of publications mentioning SF3B1 in connection with the four types of cancer increased gradually, especially for MDS and CLL, and reached peaks in 2013 at 11 and 9 articles, respectively. Regarding breast cancer and AML, their curves peaked in 2016 at 5 and 6 articles, respectively.

By comparison to the other three types of cancer, SF3B1 received the most attention in the literature as an associated MDS gene with 41 publications from 2011 to 2018, collectively. On the other hand, CLL, breast cancer, and AML are associated with 24, 20 and 15 articles, respectively. Additionally, 29 countries contributed to SF3B1 studies in link to some or all of the four types of cancer as shown in Fig. 1b. Interestingly, only 8 countries (namely, USA, Italy, France, Germany, Japan, China, South Korea, and The Netherlands) studied SF3B1 in association with all the four types of cancer. Overall, authors affiliated with United States published the highest number of articles. East Asian countries (Japan, China, South Korea, and Taiwan) studied SF3B1 in association with hematologic cancer in 24 articles and only 3 articles in link to breast cancer. Concerning European countries, the distribution is consistent among MDS, breast cancer, and CLL with 31, 30 and 27 articles, respectively, while AML-SF3B1 association received the lowest interest (8 articles).

Table 1 reports the level of attention SF3B1 has received from researchers who have studied the considered four types of cancer for the period 2007 to 2018. It lists the most studied genes by various researchers in association with the four types of cancer. From the reported results, it is obvious that SF3B1 did not receive the same level of attention from researchers. The overall effort from researchers to study SF3B1 with MDS and CLL seems significant enough in comparison with top genes as shown in Table 1. On the other hand, SF3B1 ranks after the top 10 genes for both breast cancer and AML, i.e., it has not received enough attention in the analyzed literature. Accordingly, a detailed mutation analysis was performed to find out whether SF3B1 is worthy to be studied in connection with breast cancer and AML. Most articles studied MDS and AML together as the two types of cancer have out of the top 10 genes six in common, namely JAK2, NRAS, IDH1, IDH2, DNMT3A, and RUNX1. Finally, it is worth mentioning that the analyzed literature revealed TP53 as the most studied gene in association with breast cancer and CLL.

**Table 1:** The top 10 genes associated with breast cancer, MDS, AML, and CLL based on the analyzed PubMed articles.

Breast cancer		MDS		AML		CLL	
Gene	No of PubMed articles	Gene	No of PubMed articles	Gene	No of PubMed articles	Gene	No of PubMed articles
TP53	171	JAK2	62	FLT3	469	TP53	32
PIK3CA	118	TET2	46	NPM1	159	NOTCH1	30
PTEN	54	SF3B1	38	NRAS	120	ATM	23
CDH1	48	NRAS	34	IDH2	77	SF3B1	21
AKT1	45	ASXL1	33	CEBPA	74	C11orf65	14
KRAS	45	IDH1	30	KIT	72	BIRC3	12
ESR1	41	DNMT3A	28	IDH1	68	XPO1	12
MED12	41	IDH2	28	DNMT3A	63	BRAF	11
RB1	36	RUNX1	27	JAK2	57	MYD88	9
BRAF	33	TP53	25	RUNX1	54	BTK	6
SF3B1	19			SF3B1	13		

### Numbers of mutations vary from one tissue to another

According to COSMIC, 586 somatic mutations of SF3B1 were detected in 2552 samples, originated in 30 tissues, and reported in 226 articles. The top 20 tissues were retrieved based on numbers of mutations, samples, and PubMedID, separately. By combining all outputs, soft tissue and pleura were excluded as they have low numbers of papers related to them. Similarly, salivary gland and bone were excluded as they have high numbers of related papers, but low numbers of mutations and samples. The remaining 18 tissues are listed in Table 2. Haematopoietic and lymphoid tissue is the most mutated among other tissues based on the three attributes. This is consistent with the results reported in the previous sections and in the literature. Breast, skin, lung, and large intestine are placed between 2nd and 5th with different orders based on each attribute, separately. Interestingly, the eye tissue ranked second based on the number of samples, but it was only mentioned in 9 articles. Also, urinary tract and liver have relatively high numbers of mutations and patients, however, they have not been studied enough in the literature by considering the articles analyzed in this study.

**Table 2:** The top 18 tissues associated with SF3B1 mutations based on number of mutations, samples, and PubMed articles, collectively.

Tissue	No of mutations	No of samples	No of PubMed articles
Haematopoietic and lymphoid	133	1659	99
Large intestine	98	97	14
Lung	79	83	17
Skin	66	97	18
Breast	54	97	21
Urinary tract	32	45	4
Endometrium	31	29	4
Liver	30	37	3
Central nervous system	25	22	8
Kidney	23	22	5
Prostate	21	27	11
Biliary tract	18	23	7
Eye	17	116	9
Pancreas	16	31	6
Oesophagus	14	15	8
Stomach	14	14	5
Thyroid	13	18	5
Upper aerodigestive tract	11	10	7

Data are listed in descending order based on number of mutations.

**Figure 2:**
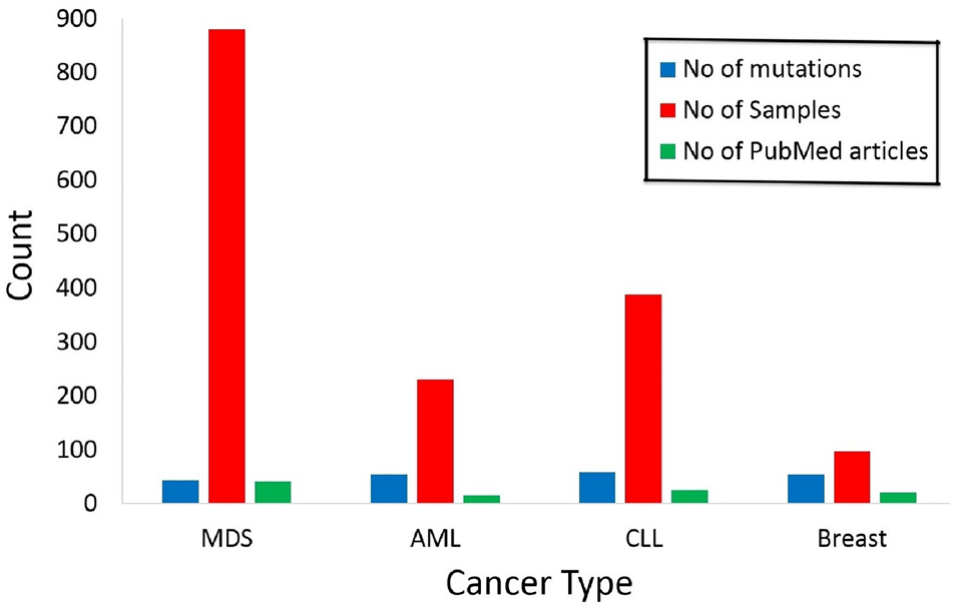
Clustered bar chart shows the distribution of SF3B1 somatic mutations in the four cancer types (MDS, AML, CLL, and BC), based on numbers of mutations, samples, and PubMed articles which are colored in blue, red, and green, respectively.

Haematopoietic and lymphoid tissue has 28 histological classifications. Among them, we were interested in MDS, AML, and CLL to compare them with BC. The frequencies of the four cancer types in terms of the three attributes we used before are shown in Fig. 2.

Among 2552 samples identified, there are 260 samples which were mentioned more than once. Their data showed that all repetition except three patients were due to multiple articles noted them and multiple mutations were found in the same tissue. “Haematopoietic and lymphoid" tissue was repeated 495 times out of 646, followed by large intestine, breast, lung, and skin that were repeated 42, 22, 14, and 12 times, respectively. These values indicate that a group of mutations may be needed to cause hematologic cancer in one patient, while fewer number of mutations can be enough to cause other cancer types such as breast, lung, and skin. The three exceptional samples which were common among haematopoietic and lymphoid are:

- One patient had p.K700E in chronic myelomonocytic leukemia (CMML) & AML associated with MDS.
- One patient had p.K700E in CMML & p.K666N in MDS
- One patient had p.K666R in MDS & p.H738Y in AML.

By definition, a hotspot mutation is the one that frequently occurs in tumor samples^18^. Accordingly, 20 hotspot mutations were identified based on the number of samples in Table 3. Additionally, we counted numbers of tissues and articles, but we did not rely on them. Overall, p.K700E is the most hotspot mutated residue (wild type lysine at 700 residue is substituted by mutant glutamic acid) from three perspectives. In addition, p.K666 is considered as a hotspot mutated residue with 6 different types of substitutions. Interestingly, p.K141K ranked second by 114 samples, but it was reported in only 4 articles. Overall, it is obvious from Table 3 that the top frequently mutated residues are located in SF3B1 HEAT repeats. These mutations may alter the HEAT domain’s confirmation, affecting SF3B1 structurally and dynamically. This may explain the reason behind not studying p.K141K, although it was detected in 114 samples. As p.K141K is located in the unstructured N-terminal, it may not have structural or functional impacts on SF3B1. These results match the experimental findings of^13,14^.

**Table 3:** The top 20 frequent mutations of SF3B1 that were detected in tumor samples.

Mutation	No of samples	No of tissues	No of PubMed articles
p.K700E	782	14	92
p.K141K	114	5	4
p.K666N	110	6	38
p.V1219V	92	3	3
p.G877G	91	2	2
p.R625H	88	9	19
p.R625C	82	11	34
p.G742D	75	6	26
p.E622D	70	5	34
p.H662Q	66	4	29
p.K666T	37	6	28
p.K666R	33	2	17
p.R625L	33	6	24
p.K666E	28	3	16
p.K666Q	20	3	16
p.H662D	17	1	11
p.K666X	17	1	3
p.G740E	16	4	12
p.Y623C	16	3	11
p.E902K	15	2	5

**Figure 3:**
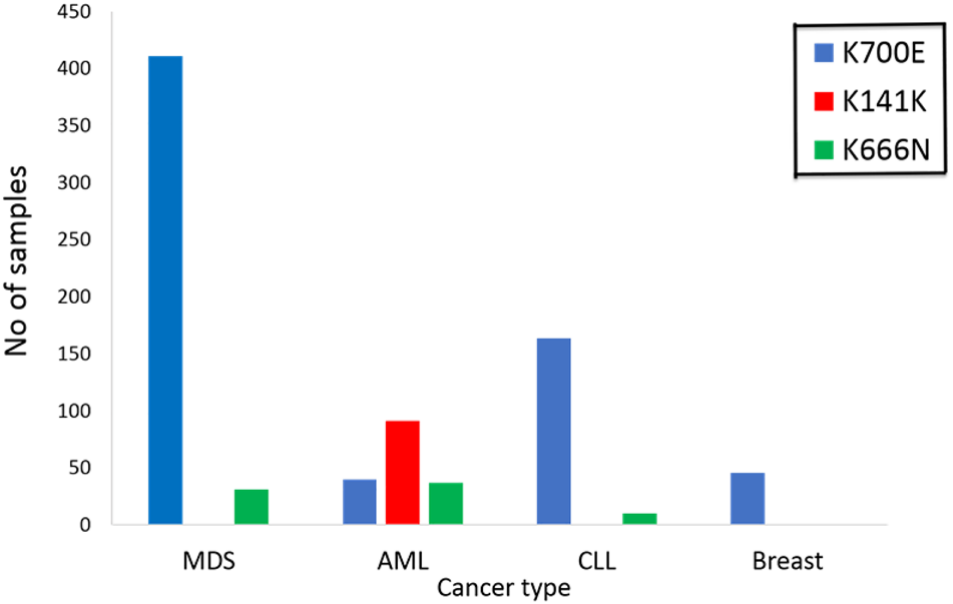
A clustered bar chart shows the number of hematologic and breast cancer patients who had any of these mutations p.K700E, p.K141K, and p.K666N, these are colored in blue, red, and green, respectively.

For detailed results, we considered the first three mutations analyzed in association with the four types of cancer. As shown in Fig. 3, among the three hotspots, breast cancer is linked to only p.K700E mutation. Although p.K141K ranks second in Table 3, it is detected in only AML patients among the four types of cancer and 20 soft tissue patients (not shown in the figure). Similarly, p.K666N is significantly linked only to hematologic cancer.

**Figure 4:**
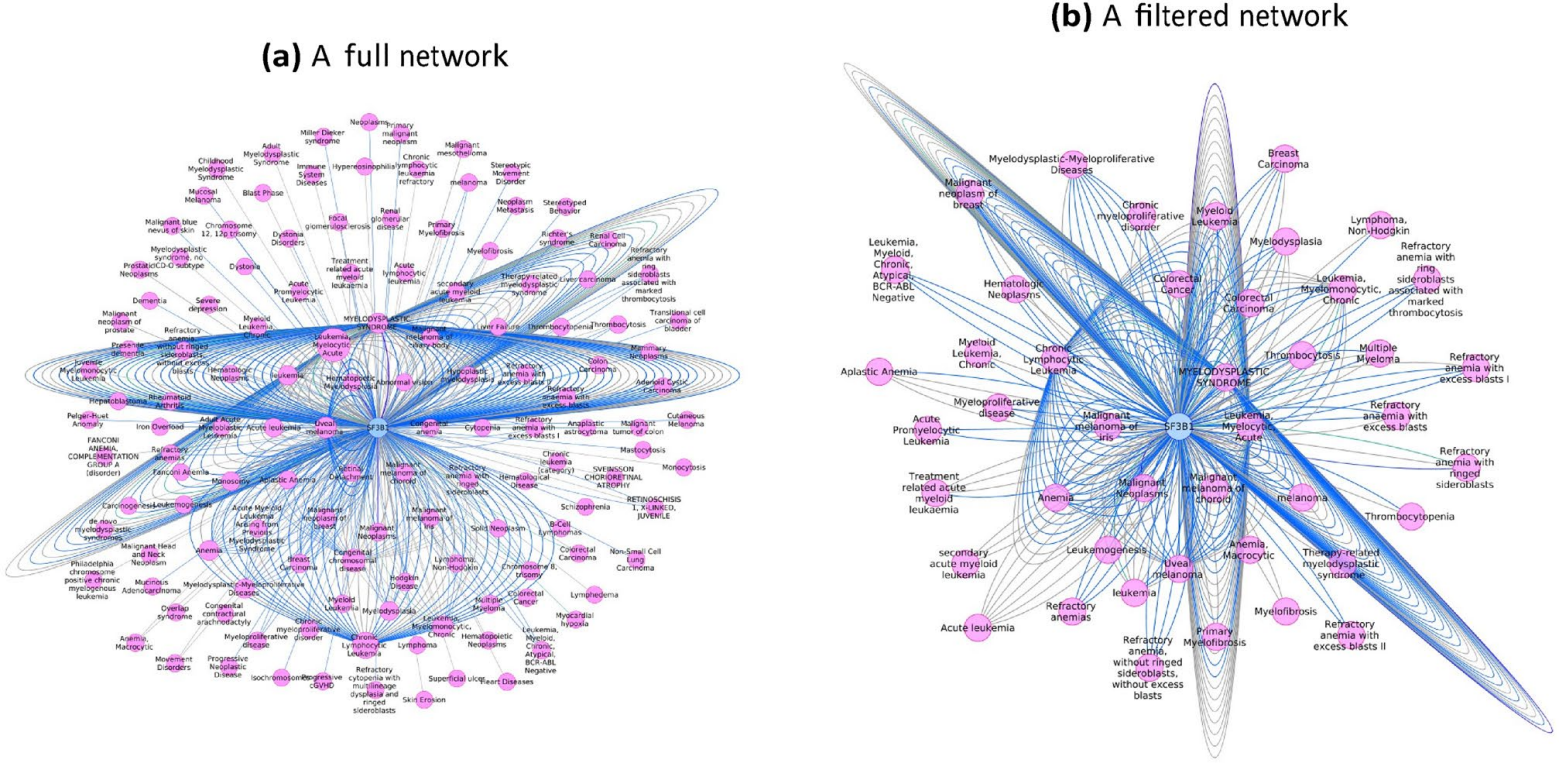
(**a**) The constructed gene-disease network consists of 131 diseases which are linked to SF3B1 by 472 edges, this list of diseases are reported from 1985 to 2018. (**b**) A filtered network including 44 diseases that are connected to SF3B1 by 349 edges. Edges are colored based on association type; blue indicates biomarker association and gray indicates causal mutation or somatic causal mutation.

**Figure 5:**
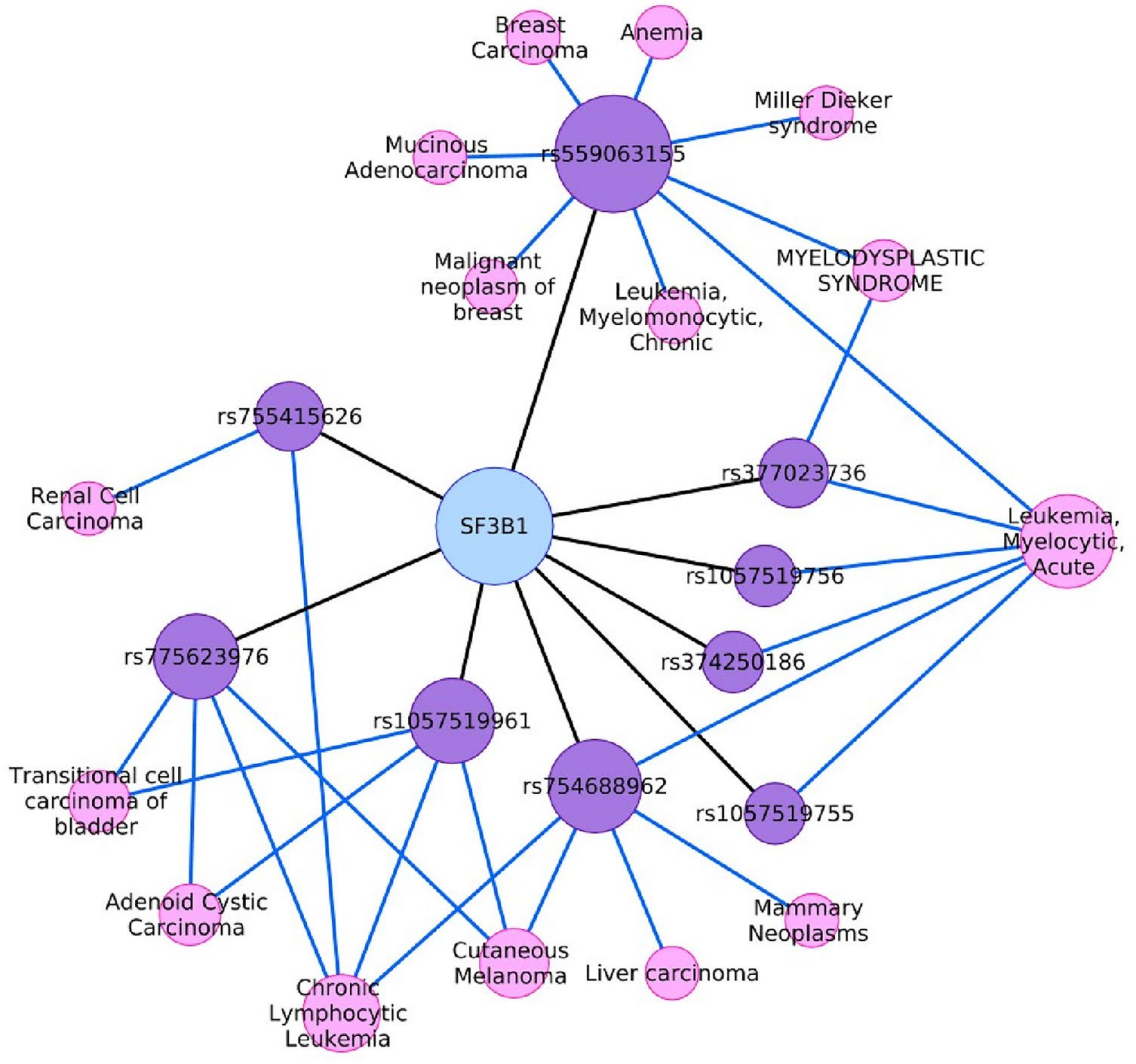
The constructed variant-disease network shows one cyan node which represents SF3B1 gene. It is connected to 9 purple nodes representing variants and each of them is connected to one or more pink nodes representing disease. Overall, the network has 25 nodes and 37 edges. Edges are colored in black or blue representing gene-variant or variant-disease associations, respectively.

The data obtained from COSMIC were confirmed from DisGeNET plugin by constructing gene-disease association and variant-disease association networks using Cytoscape. In the gene-disease network, 131 diseases are connected to SF3B1 based on (i) association type, (ii) number of associated SNPs, and (iii) studies that support each association as shown in Fig. 4a. We excluded all diseases which have a degree of one, non-cancer or hemic, and lymphatic diseases as shown in Fig. 4b. Then, we sorted the remaining list of diseases based on their degrees. MDS, AML, and CLL have the highest degree values as 84, 58, and 33 and their association scores are 0:7, 0:5, and 0:5, respectively. Then, uveal melanoma is connected by 15 edges and associated with a score of 0:6, followed by the other six hemic and lymphatic diseases. After that, malignant neoplasm of breast and breast carcinoma are placed by 6 edges for each with an association score of 0:01. In the constructed variant-disease network shown in Fig. 5, we found some consistent and some different results compared by COSMIC. K700E (dbSNP: rs559063155) ranked first by 8 connected diseases including MDS, AML, and breast. Its association score with AML is 0:7, while it is 0:01 for MDS and breast. K666N (dbSNP: rs377023736) is connected to MDS and AML with 0:7 association score for each. Interestingly, K141 does not exist in the DisGeNET database. This may indicate that it is not a pathogenic mutation, or it has not been reported with clinical impact.

### Individual gene vs. group gene based studies of SF3B1-diseases association

To further investigate the connection between SF3B1 and the considered four types of cancer based on confirmed information already stored in the existing repositories, the association of each gene group that includes SF3B1 and the four targeted cancer types were identified using publicly available sources, namely COSMIC and DisGeNET. The results obtained from COSMIC and DisGeNET are reported in Tables 4 and 5, respectively. For comparison, we added the corresponding data of SF3B1 individually. First, COSMIC data in Table 4 shows that the highest number of patients who carry mutant genes from any of the three families were diagnosed with breast cancer. We also compared four cancer types with respect to one family and found that ARMH genes affect the highest number of patients with breast cancer, CLL, and AML. The exception is MDS related to which more patients carried mutant spliceosome genes than other families. For B-WICH family, it contributes the least to all of them because it is the smallest family that contains 8 genes including SF3B1.

**Table 4:** The association between each of the four diseases and SF3B1 or its family separately based on COSMIC database.

	SF3B1	ARMH (243)	Subcomplex of major spliceosome (69)	B-WICH (7)
Breast cancer				
No of genes	1	235	65	7
No of mutations	51	49,330	532	105
No of samples	113	2534	821	232
MDS				
No of genes	1	38	6	1
No of mutations	44	76	21	1
No of samples	89	91	242	2
AML				
No of genes	1	218	46	7
No of mutations	44	450	68	9
No of samples	135	593	248	16
CLL				
No of genes	1	215	47	7
No of mutations	57	500	34	5
No of samples	378	597	108	18

**Table 5:** The association between each of the four diseases and SF3B1 or its family separately based on DisGeNET and degree centrality.

	SF3B1	ARMH	Subcomplex of major spliceosome	B-WICH
Breast cancer				
No of associated genes	1	36	9	5
Degree	6	402	16	17
MDS				
No of associated genes	1	9	3	1
Degree	84	21	37	2
AML				
No of associated genes	1	15	5	2
Degree	58	55	18	29
CLL				
No of associated genes	1	9	3	1
Degree	33	118	4	2

**Figure 6:**
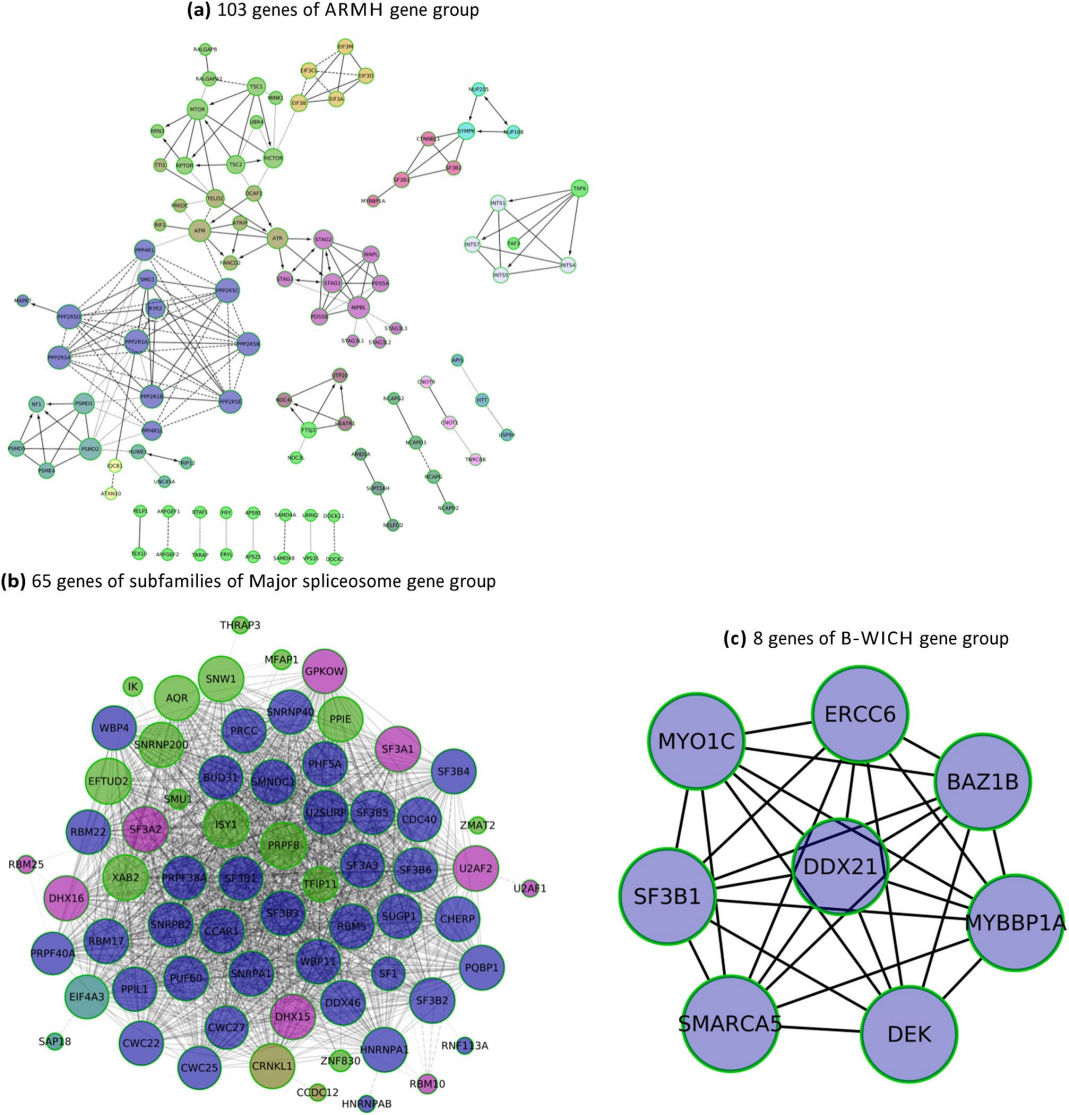
(**a**) The constructed gene network contains 103 genes of ARMH family and 188 edges. They compose 16 components where the giant one has 56 genes and the second has 7 genes including SF3B1; they are linked to 10 genes including SF3B1. (**b**) One component of 65 genes of some subfamilies of major spliceosome root family and 1321 edges. (**c**) One component of 8 genes of B-WICH family and 28 edges. Edge type is mapped to the FI direction attribute values; "- > " for activate/catalyze, " < -" for activated by/catalyzed by, " <-> " for catalyze/catalyzed by, "-|" for inhibition/dephosphorylation, solid line for FIs extracted from complexes or inputs, and dashed line for predicted FIs.

Reactome FI plugin in Cytoscape was utilized to perform gene set analysis and highlight the interactions between SF3B1 and each family by constructing three different gene networks. We found the following results:

1. Armadillo-like helical domain: As shown in Fig. 6a, SF3B1 has only 4 edges out of 188 edges of the whole network. It is linked to CTNNBL1, SYMPK, MYBBP1A, and SF3B2. This weak connection to other genes occurs because ARMH genes share a structural property with a different function for each gene^19^.
2. Subcomplex of Major spliceosome: It is a dense network in which SF3B1 has 50 edges and the maximum degree value is 53 as shown in Fig. 6b.
3. B-WICH chromatin-remodelling complex subunits: SF3B1 has 7 edges, same like all other nodes as shown in Fig. 6c. Functional similarities in this network and major spliceosome network form more edges between SF3B1 and other genes.

We confirmed COSMIC data by deriving a gene-disease association network for each family. The data reported in Table 5 show that breast carcinoma has the highest number of associated genes in each family’s network. However, it is connected to the highest number of edges only in ARMH network and the lowest number in B-WICH and spliceosome networks. Numbers of edges that connect ARMH genes to Breast cancer, CLL, and AML are the highest among other families. On the other hand, MDS has the highest degree with spliceosome genes. B-WICH family ranks second for breast cancer and AML (based on a number of edges) ranks the lowest for MDS and CLL with two edges for each of them.

The data reported in Tables 4 and 5 indicate that mutations of SF3B1 may drive MDS and CLL as the number of patients who carried mutant SF3B1 is by far higher than the number of patients who carried other mutant splicing factors. On the other hand, contribution of other splicing factors moderately increase the number of patients, who were diagnosed with AML, and dramatically increase for breast cancer patients. The least affected pathway in all diseases is RNA polymerase II transcription that is carried out by B-WICH complex genes followed by splicing mechanism in all diseases except MDS. Biological mechanisms that involve ARMH genes seem to be the most driven even for breast cancer, followed by CLL, AML, and MDS the BC.

**Figure 7:**
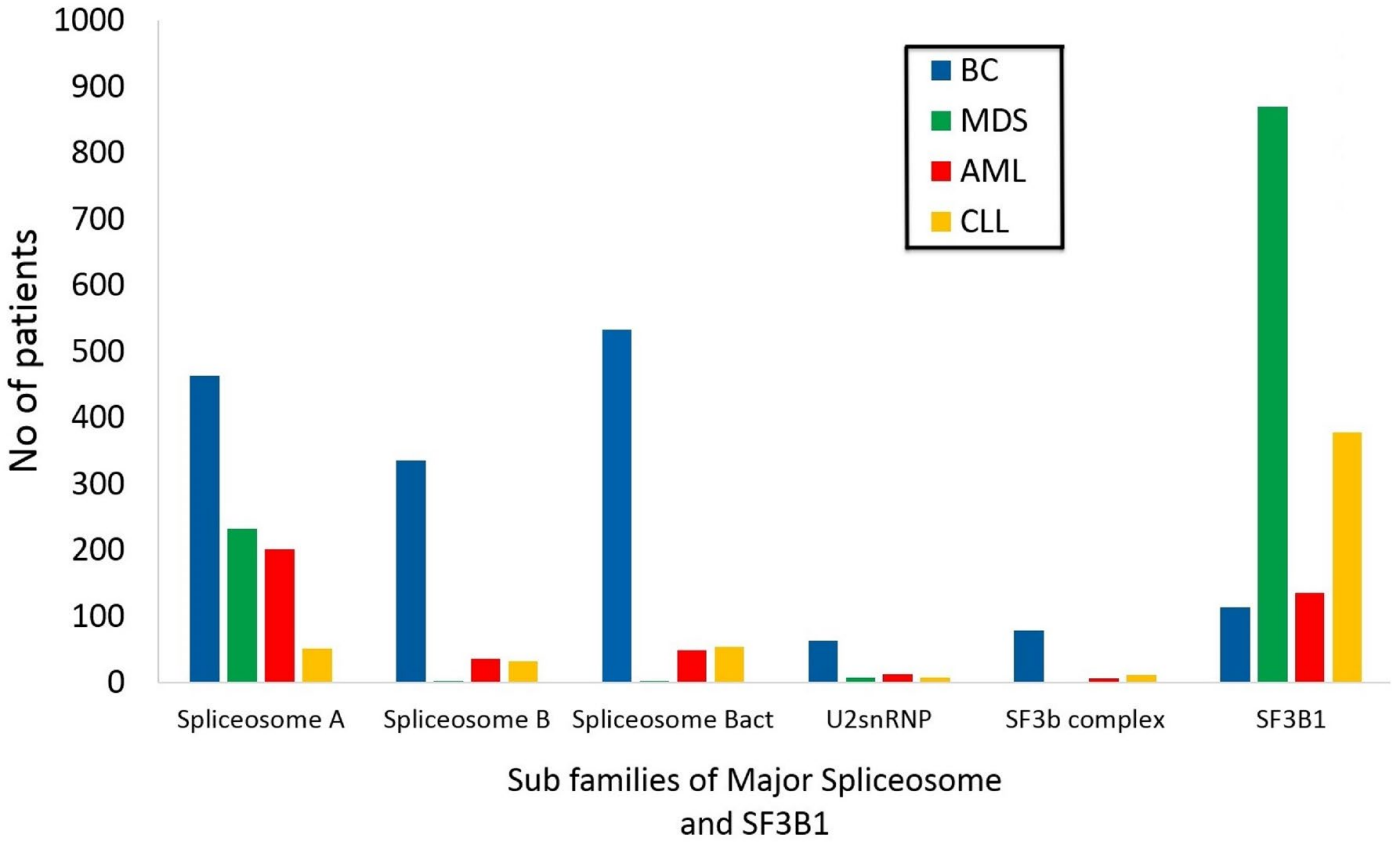
A clustered bar chart shows the contribution of each subgroup of major spliceosome gene group to BC, MDS, AML, and CLL, these are colored in blue, green, red, and yellow, respectively.

To support the results found using the literature analysis, the COSMIC database was used to retrieve the mutations associated with the discovered genes. As mentioned before, “Major spliceosome" family has six subgroups, five of which have “U2 small nuclear ribonucleoprotein" as a subgroup that contains “SF3b complex". We included spliceosomal A complex, spliceosomal B complex, spliceosomal Bact complex, U2 small nuclear ribonucleoprotein, and SF3b complex. For more detailed results, we reported the number of patients who had mutant genes from each subfamily of major spliceosome as shown in Fig. 7. By comparison, SF3B1 is the most frequently mutant gene in the four diseases; however, SF3b complex is not the highest mutant subgroup in all diseases. For instance, spliceosome Bact complex is the most frequent subgroup associated with breast cancer. While spliceosome A complex is the most associated family with AML. The analyzed data refer to mutant genes which are more crucial in the development and/or prognosis of each of the four cancer types.

### Impact of aberrant splicing on other pathways

As described in Section of the methodology, some queries have been coded to check how the four types of cancer are interrelated by considering SF3F1 mutations. To achieve this, we coded a number of queries by considering combinations of the four types of cancer. For each one of the four types of cancer, we retrieved number of patients who have SF3B1 mutations in common with each of the remaining three types of cancer. In other words, for each cancer type, we run three queries to find out patients who have been classified as having the two cancer types to extract repeated samples that have SF3B1 mutations in one disease. The coded DB4S queries resulted in the following results:

1. When SF3B1 is mutant in MDS:
  There are 6 common patients with AML.
  No common patients with CLL.
  There are 9 common patients with breast cancer.
2. When SF3B1 is mutant in AML:
  No common patients with MDS, CLL, or breast cancer.
3. When SF3B1 is mutant in CLL:
  There are 2 common patients with MDS.
  No common patients with AML.
  There are 5 common patients with breast cancer.
4. When SF3B1 is mutant in breast cancer:
  No common patients among MDS, AML, and CLL.

The returned numbers of samples from each query are low. However, these results may indicate that aberrant splicing due to SF3B1 mutations may lead to developing (i) MDS that may be followed by developing AML or breast cancer, (ii) CLL that may be followed by developing breast cancer or MDS in extremely rare cases, and (iii) AML or breast cancer without prior cancer formation. It is impossible to figure out which tumor was formed first; however, these findings are important to understand more about metastatic cancers. MDS may be firstly formed due to aberrant splicing which produces abnormal transcripts leading to AML or breast cancer development. The other possibility is that the formation of breast tumor is prior to MDS or AML. As reported in the literature, this case may occur due to chemo/radio therapies which are commonly used to treat breast cancer^20^. They may alter or damage DNA content, producing more mutant genes and consequently leading to MDS or AML development.

**Figure 8:**
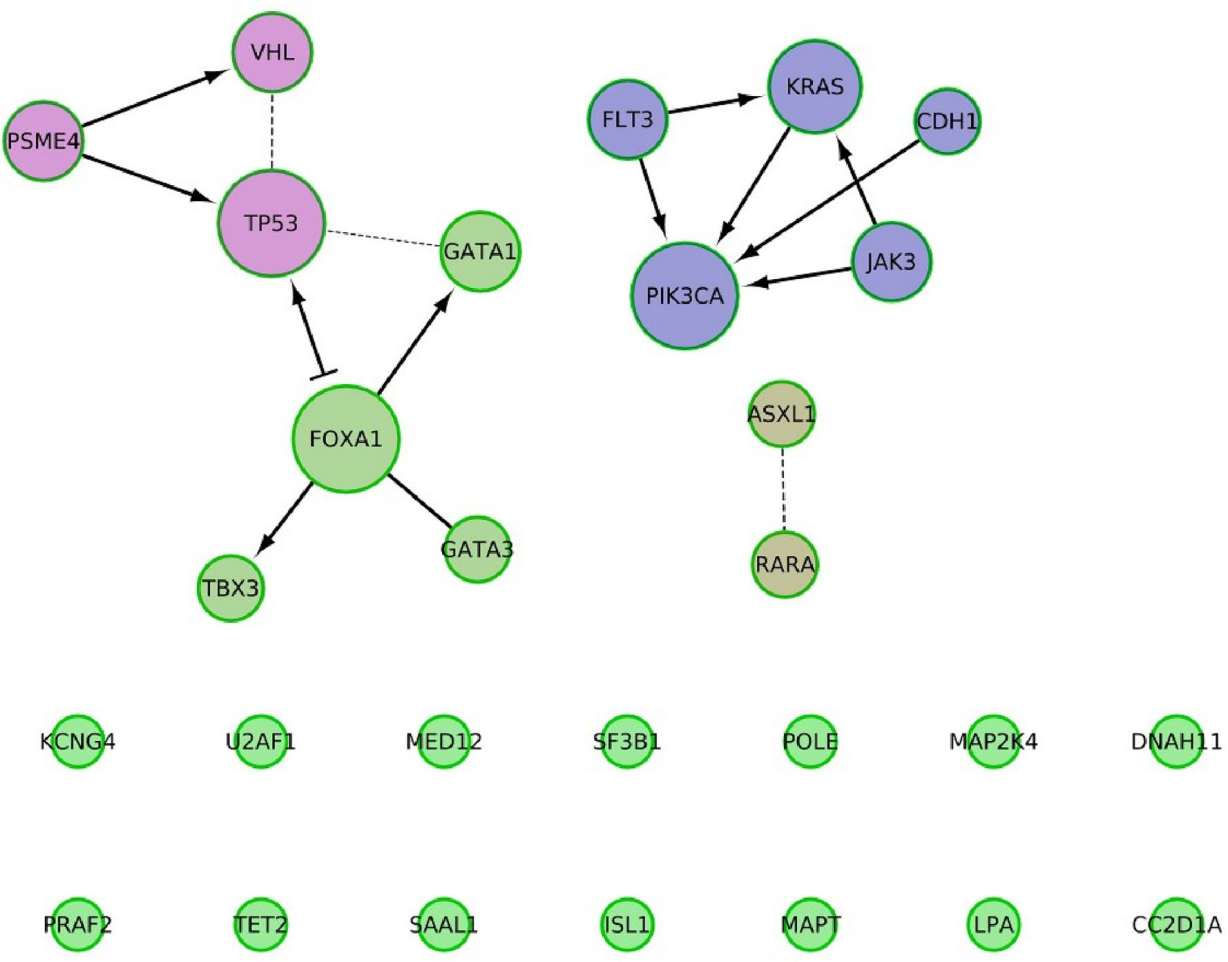
The constructed gene network by Reactome FI plugin in Cytoscape shows in total 28 nodes and 15 edges. The network consists of 3 connected components. The giant one contains 7 nodes and two small components consist of 5 and 2 nodes. There are 14 disconnected nodes. Edge’s size represents FI score and line type represents FI direction as previously described. Node’s size reflects its degree and node’s color is based on FI clustering.

As reported in Fig. 8, the corresponding list of genes, which are carried by the repeated samples, was analyzed by Reactome FI to investigate the impact of aberrant splicing on other pathways. We found 7 genes participate in the pathway of RNA Polymerase II Transcription and other pathways are listed in Table 6.

**Table 6:** Reactome FI analysis for the gene network displayed in Fig. 8.

Common pathways	No of genes	Genes from the network
Pathways in cancer (K)	8	FLT3, CDH1, VHL, JAK3, PIK3CA, RARA, KRAS, TP53
RNA polymerase II transcription (R)	7	GATA3, GATA1, MED12, PSME4, RARA, KRAS, TP53
Signaling pathways regulating pluripotency of stem cells (K)	5	JAK3, ISL1, TBX3, PIK3CA, KRAS
Human T-cell leukemia virus 1 infection (K)	5	JAK3, MAP2K4, PIK3CA, KRAS, TP53
MAPK signaling pathway (K)	5	FLT3, MAP2K4, KRAS, MAPT, TP53
PI3K-Akt signaling pathway (K)	5	FLT3, JAK3, PIK3CA, KRAS, TP53

Table 6 provides insights into the impact of aberrant splicing on other pathways. The produced abnormal spliced transcripts encode to proteins which are involved in vital pathways, mitogen-activated protein kinase (MAPK) and PI3K-Akt signalling pathways. Associated biological processes are regulation of gene transcription, cell proliferation, and apoptosis. Misregulation of MAPK and/or PI3K-Akt signaling cause uncontrolled cell proliferation and resistance to apoptosis of tumor cells^21–23^. Additionally, positive and/or negative regulation of transcription by RNA polymerase II indicate the connection between mRNA splicing and transcription by polymerase II as reported before in an experimental study^24^.

### Relation between hematologic malignancies and breast cancer

**Figure 9:**
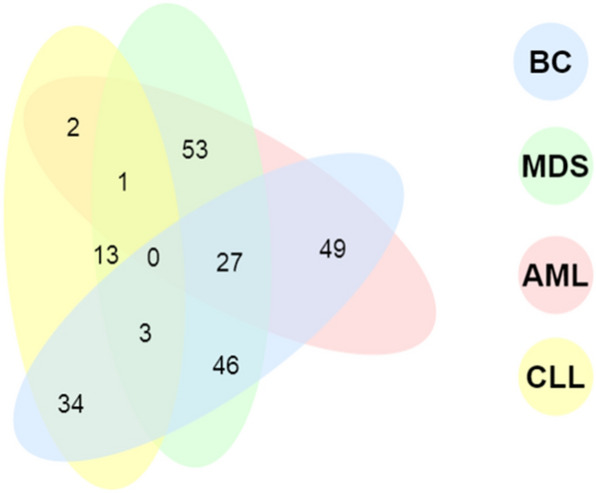
Venn diagram shows the number of common patients among each possible combination within the four cancer types. All data are reported except the number of patients having AML, CLL, and BC as zero.

Interactions within hematologic malignancies or between any of them and breast cancer were investigated. Similar queries to those described in the previous subsection were executed without specifying any gene name. Inner join of each combination resulted in a number of overlapping patients as illustrated in Fig. 9.

The values reported in Fig. 9 are relatively higher than those reported in the previous section because all genes were included regardless of the conditions. Predicting relations among the four cancer types can be possible from these results. For instance, MDS and AML have the most significant relation as there are 53 patients who had both diseases. It is the highest number of common patients among all cancers. Also, the relations between each of the three type of cancer MDS/AML/CLL and breast cancer seem to be significant and among the three types as well since 27 patients were diagnosed by these three cancer types. On the other hand, only one patient was common among the three hematologic cancer types and 3 patients were common among MDS, CLL, and breast. Regarding each two joined tables, the number of common patients between CLL and AML/MDS was low, while it was relatively high for CLL and BC.

Additionally, these results suggest that developing breast cancer needs higher number of mutant genes association with CLL/AML, which are 1014 and 247, respectively. 159 associated genes which are mutant in the same samples had BC. This is just below the highest number of genes in MDS common patients with AML (162). Another interesting finding is that only 16 genes have been identified in MDS patients, while 420 CLL-associated genes were witnessed in the same patients.

**Figure 10:**
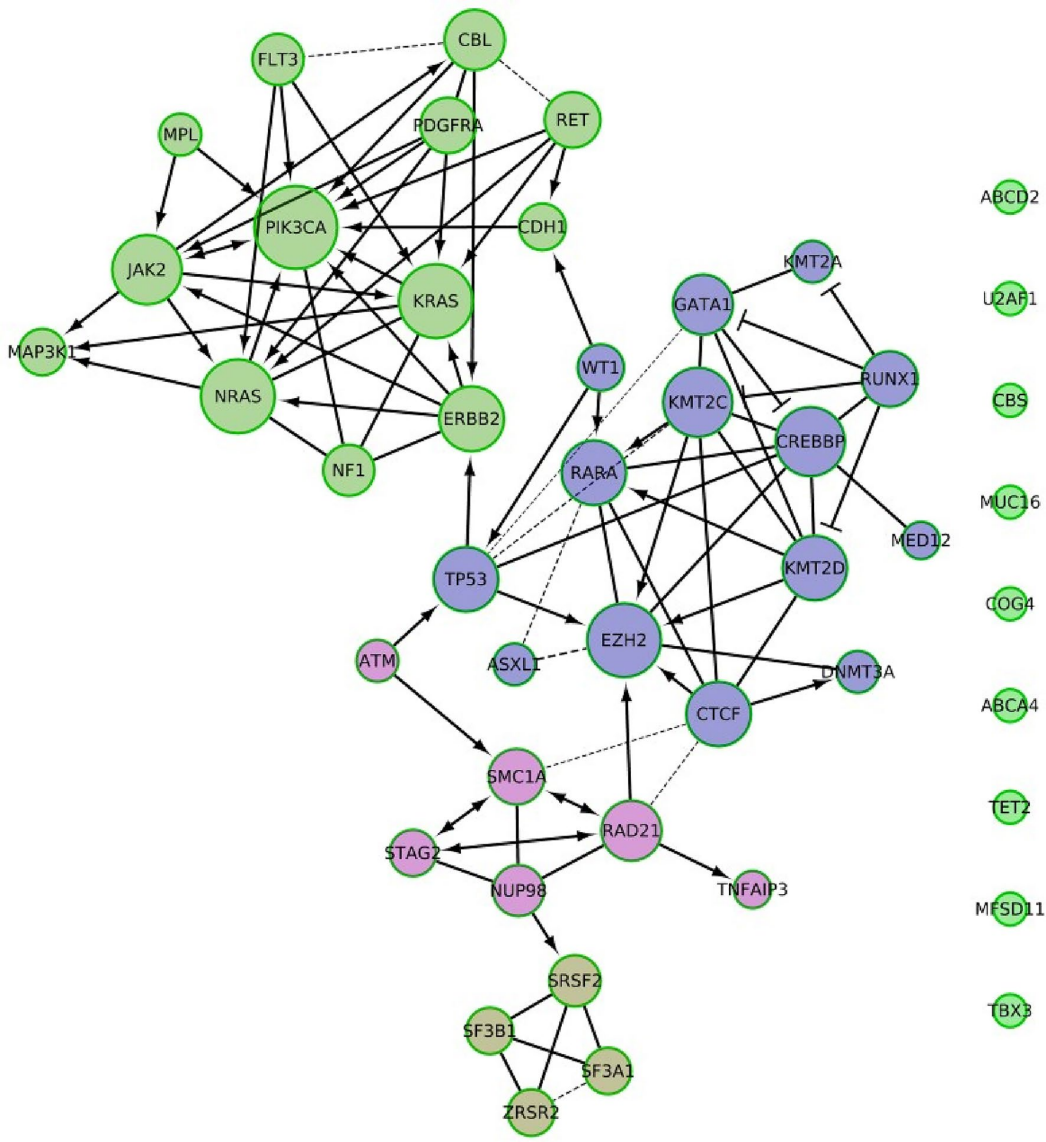
The constructed gene network by Reactome FI plugin in Cytoscape shows in total 42 nodes and 92 edges. The network consists of one giant connected component which contains 37 nodes and 9 disconnected nodes. Edge’s size is mapped to FI score and line type is mapped to FI direction as previously described. Node’s size reflect its degree and nodes’ colors are based on FI clustering.

The top mutant genes were extracted from each query, combined and filtered based on the number of repetitions to extract only frequently repeated genes as shown in Fig. 10. In this section, more genes were retrieved from COSMIC and added to the network shown in Fig. 10, and more interactions among genes were formed. For instance, SF3B1 was a disconnected gene in the constructed gene network shown in Fig. 8. Unlike the network shown in Fig. 10, SF3B1 is connected to 3 genes which are connected to others, forming one connected component. There are multiple paths which connect SF3B1 to TP53 (top associated gene to CLL and breast cancer). The relationship between SF3B1 and other genes involved in other pathways can be anticipated from the network shown in Fig. 10. Additionally, away from SF3B1, there is a direct connection between TP53 and another cluster of genes containing JAK2 and FLT3, the top associated genes to MDS and AML, respectively. Pathway enrichment analysis was conducted for this gene network to obtain common pathways. We found (i) RNA Polymerase II transcription, (ii) microRNAs in cancer, and (iii) central carbon metabolism in cancer, as the three most common pathways having the highest numbers of genes as listed in Table 7.

**Table 7:** Reactome FI analysis for the gene network displayed in Fig. 10.

Common pathways	No of genes	Genes from the network
Pathways in cancer (K)	14	RET, FLT3, CBL, NRAS, CDH1, ERBB2, JAK2, PDGFRA, CREBBP, RUNX1, PIK3CA, RARA, KRAS, TP53
RNA polymerase II transcription (R)	14	KMT2D, KMT2A, KMT2C, GATA1, MED12, ERBB2, CREBBP, RUNX1, RARA, SRSF2, KRAS, ATM, TP53, EZH2
MicroRNAs in cancer (K)	10	NRAS, ERBB2, PDGFRA, CREBBP, DNMT3A, PIK3CA, KRAS, ATM, TP53, EZH2
Central carbon metabolism in cancer (K)	8	RET, FLT3, NRAS, ERBB2, PDGFRA, PIK3CA, KRAS, TP53
SUMOylation (R)	8	RAD21, CREBBP, DNMT3A, SMC1A, STAG2, RARA, NUP98, TP53
RAF/MAP kinase cascade (R)	8	RET, FLT3, NRAS, ERBB2, JAK2, PDGFRA, NF1, KRAS
MAPK signaling pathway (K)	8	FLT3, NRAS, ERBB2, PDGFRA, MAP3K1, NF1, KRAS, TP53
PI3K-Akt signaling pathway (K)	8	FLT3, NRAS, ERBB2, JAK 2, PDGFRA, PIK3CA, KRAS, TP53

Although the splicing mechanism is not fully understood yet, experimental studies identified potential drugs that target SF3B1. For instance, the study conducted by Obeng et al.^25^ in 2016 reported that hematopoietic stem and progenitor cells (HSPCs) expressing SF3B1^+=K700E^ showed an increased sensitivity to E7107 in vitro and in vivo compared to SF3B1^+=+^. E7107 is a derivative of the Pladienolide family of a natural product that has been shown to have antitumor activity and inhibitory effect against spliceosome assembly. Later, Seiler et al.^26^ reported that H3B-8800 showed more potency to bind and inhibit splicing catalysis by both wildtype and mutant SF3B1 in vitro, compared to E7107. Moreover, treatment with H3B-8800 resulted in lethal activity only in mutant SF3B1 pancreatic cancer cell lines, while E7107 did not show any clear effects on the same cell line. Recently, a study by Gama-Brambila et al.^27^ reported potential results for targeting SF3B1 by the proteolysis-targeting chimeras (PROTACs). Fusion of thalidomide into structure of OCT4-inducing compound 2 (O4I2) resulted in PROTAC-O4I2 that selectively degraded SF3B1 and induced cellular apoptosis in K562 cells and in Drosophila intestinal tumor model.

## Conclusion

SF3B1 has a crucial role in the splicing mechanism as it recognizes and selects the branch splice site along with other subunits of splicing machinery. As described in this article in association with the studied four types of cancer, SF3B1 is commonly mutated in many cancer types and these associated mutations cause aberrant splicing, production of abnormal transcripts, and cancer development^4–6^.

**Figure 11:**
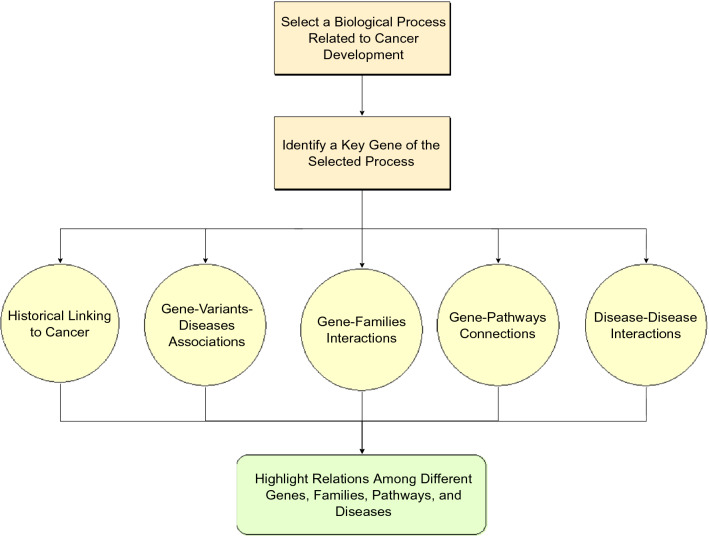
The block diagram depicting the work flow of the conducted study.

We utilized publicly accessible databases and open source tools for network construction and analysis to demonstrate an analytical view about SF3B1 based on a set of articles from Pubmed where SF3B1 is associated with the four types of cancer considered in this study. We limited the study to articles published between 2007 and 2018. As a result of the conducted investigation, we realized that SF3B1 is association with hematologic malignancies, represented by (MDS, AML, and CLL) vs. BC. The criteria we applied in each phase of our framework which is depicted in Fig. 11 could consider both strong and weak associations between SF3B1 and various types of cancer through a set of genes and cellular pathways. Accordingly, our findings help researchers conduct more efficient and precise experiments to study the relation among mutant SF3B1, aberrant splicing, and cancer development. For instance, SF3B1 is highly relevant to blood cancer and can be studied solely as a therapeutic target. In contrast to breast cancer, SF3B1 is less relevant and more splicing factors may be considered to reveal significant results. Additionally, functional consequences of aberrant splicing were analyzed and showed that it leads to abnormalities in other vital processes such as regulation of cell proliferation, apoptosis, and transcription by RNA polymerase II. These conclusions, though need experimental validation, may help developing new therapeutic approaches that target multiple pathways in cancerous cells. However, our findings reported in this study are limited to the outcome from the analysis of the located articles; and hence we cannot generalize any findings to comment on other connections of SF3B1 which may be very significant and may be thoroughly investigated in another study.

Finally, relations among different types of cancer (MDS, AML, CLL, and BC) have been investigated. The significant relations we found were between MDS & AML, each of MDS, AML, CLL, and BC separately, and MDS, AML, and BC collectively. Further research is needed to confirm these relations and to provide more insights at the molecular levels. Consequently, it may help develop new approaches to prevent transformation of cancer from one type to another. We want to extend this study by considering a longer period and more cancer types in addition to other diseases and mutations associated with SF3B1. Our target is to reveal more associations which could be tested in the wetlab by our collaborators from the medical domain.

## Methods

Our methodology described in this paper consists of the steps depicted in Fig. 11. It is a general framework which may be applied to any cancer related cellular process. In this study, we selected pre-mRNA splicing as a case study for the proposed methodology. Then, among many splicing factors, our analysis shed light on SF3B1 since it is reported in the literature as the most frequently mutated component of SF3b protein complex in many types of cancer^14^. Our analysis approach is mainly divided into five phases that are represented by yellow circles in the block diagram shown in Fig. 11.

It is important to emphasize that publicly accessible databases have been used in this study, and hence all methods were performed in accordance with the relevant guidelines and regulations. In order to collect the needed data for constructing and analyzing different gene networks, the following accessible databases and tools were used in our study:

1. March and September 2019 releases of the Catalogue of Somatic Mutations in Cancer (COSMIC)^28^: to collect somatic mutation data, including many attributes such as tissue types, nucleotide and amino acid sequence changes, mutation’s types, PubMed PMID of studies reporting each mutation, sample names, etc.
2. The HUGO Gene Nomenclature Committee (HGNC)^29^: to get a list of genes that have the same family.
3. DB Browser for SQLite (DB4S): to import tables of COSMIC data and execute SQL queries for data filtering, processing, and combination^30^.
4. Cytoscape: an open source bioinformatics software platform to build and visualize gene–gene interactions and genes-diseases interactions networks. Also, we used some features that are available in Cytoscape as plugins^31^ such as:
  - Reactome FI: a Functional Interaction Network to identify common pathways, biological processes, and molecular functions for a set of genes^32^.
  - DisGeNET: to build gene-diseases association networks^33^.
  - NetworkAnalyzer: for subnetwork creation and network analysis by calculating topological parameters and centrality measures^34^.

### Investigating SF3B1 somatic mutations frequencies and the related diseases

SF3B1 somatic mutations were detected in many cancer types^14^. Accordingly, COSMIC mutation raw data of SF3B1 were processed and analyzed by DB4S to (i) compare all affected tissues, (ii) list associated mutations with high rates, and (iii) show the distribution of hotspot mutations in all affected tissues. COSMIC reports 37 attributes in each table. Among them, five attributes (AAmutation, SampleName, PubmedId, PrimaryTissue, and HistologySubtype1) were counted using the “COUNT" clause. The returned values represent statistical parameters to evaluate and compare different mutations. COSMIC classified all hematologic cancers in one tissue, namely “Haematopoietic and lymphoid". Therefore, histological classification was used to report specific data for MDS, AML, and CLL, separately.

To confirm these outcomes, DisGeNET was used to identify highly associated diseases. These parameters were used to evaluate DisGeNET data:

1. Gene-disease association (GDA) score in the range [0,1]; it is computed by DisGeNET database^35^ based on the number and type of sources (level of curation, organisms), and the number of studies which reported the specific association.
2. Similarly, variant-disease association (VDA) score was considered for variant nodes, but it is computed based only on numbers of sources and publications.
3. Disease’s degree that is calculated by NetworkAnalyzer. Higher degree means higher number of edges connect a gene or variant node to a disease node, indicating higher number of evidence reporting the association between gene/variant and disease.

### Identifying linked families to SF3B1

Each gene belongs to at least one family that contains a group of genes generated from a common ancestral gene. In some cases, the whole family is associated with a single disease. Accordingly, the associations of each gene in the family of SF3B1 with breast and hematologic cancer types were investigated and compared with the obtained results from the previous analyses of gene-based data. As reported in the HGNC database, SF3B1 is a member of three families:

1. Armadillo-like helical domain containing (ARMH): 244 genes have a common superhelical structure, but they have different functions^36^.
2. SF3b complex: 7 genes form the multi-component SF3b complex to recognize the branch point of pre-mRNA for splicing. SF3b complex is also a subgroup of “U2 small nuclear ribonucleoprotein” which has a root family of “Major spliceosome". There are 145 genes by considering all subfamilies of “Major spliceosome".
3. B-WICH chromatin-remodelling complex subunits (B-WICH): 8 genes are involved in the mechanism of regulating RNA Polymerase III Transcription^37^.

Two approaches were used to test the effect of including other genes from the same family in the association with any cancer type. First, we accessed HGNC database to obtain two lists of genes from ARMH and B-WICH families. For major spliceosome, we did not include all genes since the cryo-EM study demonstrated that SF3b complex is disassociated after the late B^act^ state^38^. Spliceosomal C and spliceosomal P complexes formed after B^act^ state. In addition, spliceosomal E complex is a superfamily of “U1 small nuclear ribonucleoprotein”. Therefore, we excluded these three families. The final list included 244 ARMH genes, 8 genes from B-WICH complex, and 70 spliceosome components. Then, we merged each family’s list with COSMIC tables of MDS, AML, CLL, and BC separately. The numbers of mutations and samples were retrieved for a comparison with the resulted values associated only with SF3B1.

In the other approach, Reactome FI app was used to derive a gene network of each family and highlight common pathways among its genes. Then, a gene-disease network was derived using DisGeNET app for each family network. Based on the number of associated genes and disease’s degree parameters, we could determine whether including genes from any of the three families would increase the association with the considered cancer types. As explained in detail in the results section, adding genes, which have the same origin, increased the association with specific cancer types.

### SF3B1-pathways connections

Aberrant splicing causes a functional impact on other cellular pathways that lead to cancer development^39^. To figure out which pathways could be affected by SF3B1 mutations, we searched for patients who carry mutations associated with SF3B1 and other genes in the same disease. The query returned an empty result, i.e., we did not find any patient with the specified condition. Then, we searched for all BC, MDS, AML, and CLL patients who carry SF3B1 mutation in one disease and have other genes in another disease. The executed queries were based on: (i) “INNER JOIN" clause to select the common “sample_name" attribute between two tables. (ii) ‘WHERE" clause to specify that “gene_name" attribute is SF3B1 in one of the two diseases. Lists of mutant genes carried by the overlapping samples among cancer types were retrieved and mapped into Cytoscape to find common pathways using Reactome FI.

### Disease–disease interactions

In order to investigate existing interactions between subtypes of hematologic cancer and/or with breast cancer, queries similar to the ones in the previous section were executed without a specific gene’s name. By inner joining every two tables, we retrieved two lists of mutant genes carried by common patients where each list corresponds to one disease. For more significant results, we combined all the lists of genes and neglected any gene that was mentioned only once. Then, a distinct gene list was mapped into Cytoscape to find common pathways using Reactome FI.
